# Phenylalanine regulates initiation of digestive enzyme mRNA translation in pancreatic acinar cells and tissue segments in dairy calves

**DOI:** 10.1042/BSR20171189

**Published:** 2018-01-25

**Authors:** Long Guo, Huibin Tian, Jing Shen, Chen Zheng, Shimin Liu, Yangchun Cao, Chuanjiang Cai, Junhu Yao

**Affiliations:** 1 College of Animal Science and Technology, Northwest A&F University, Yangling, 712100, China; 2 UWA School of Agriculture and Environment, The University of Western Australia, Crawley, WA 6009, Australia

**Keywords:** α-amylase, dairy calves, mammalian target of rapamycin (mTOR), pancreas, phenylalanine, translation regulation

## Abstract

As new nutritional strategies for ruminant are designed to change production efficiency by improving the supply of rumen protect protein, lipid, and even starch, the digestive system must fit to utilize these increased nutrient supplies, especially the pancreas. The objective of this study was to investigate the effects of phenylalanine (Phe) on digestive enzymes synthesis or secretion and cellular signaling in pancreatic acinar (PA) cells of dairy calves. The PA cells isolated from fresh pancreas of dairy calves, and cultured in completed RIPA 1640 medium with no fetal serum but 0, 0.15 and 0.45 mM Phe at 37°C in CO_2_ incubator for 120 min. The pancreatic tissue segments (PTS) was cut approximately 2 × 2 mm from the fresh pancreas, and incubated in oxygenated Krebs-Ringer bicarbonate (KRB) buffer containing 0 or 0.35 mM Phe at 39°C for 180 min, and the samples were collected every 60 min after incubation. In PA cells, Phe increased (*P* < 0.05) the α-amylase secretion and mRNA expression, the phosphorylation of ribosomal protein S6 kinase 1 (S6K1) and eukaryotic initiation factor 4E binding protein 1 (4EBP1). In PTS, the Phe increased (*P* < 0.05) α-amylase and trypsin synthesis, secretion and mRNA expression, as well as the phosphorylation of S6K1 and 4EBP1. Conclusively, these results suggested that Phe regulates the synthesis or secretion of α-amylase, trypsin and lipase through mRNA translation initiation factors – S6K1 and 4EBP1.

## Introduction

Digestive enzymes are necessary part of the chemical digestion process for animals, and excreted from exocrine glands (e.g., salivary gland, pancreas, etc.) of cells in the gastrointestinal mucosa [1]. These enzymes include amylase, lipase, and protease for carbohydrates, fats, and proteins digestion respectively, are produced by pancreatic acinar (PA) cells (pancreatic exocrine cells). For ruminant, the digestion and utilization of nutrients, especially starch, may be limited and these limitation could be caused by digestive enzymes inadequate synthesis and release into the small intestine [2,3]. So, regulation of ruminant pancreatic enzyme synthesis and secretion is an important and worthwhile work for improving both meat and milk yield in ruminant production.

Recently, more and more researchers interest in amino acids (AAs) nutrition to improve animal production efficiency [4]. The results from study revealed that potential for the specific and independent regulation of enzyme secretion by digestive end products of glucose and especially AA [5]. L-Phenylalanine (Phe) is a nutritionally essential amino acid (EAA) and has many physiological function [6]. Phe has been reported to enhance the synthesis and release of pancreatic enzymes [5,7,8]. But the mechanism of actions is not clear.

In a variety of mammalian cells (mammary epithelial cells, skeletal muscle cells, adipocytes, etc.), AAs, especially branch-chain amino acids (BCAAs), influence the phosphorylation state and function of a number of proteins referred to mRNA translation that are regulated via the rapamycin-sensitive mammalian target of rapamycin (mTOR) signaling pathway [9]. This signaling pathway controls the protein synthesis of cells [10] and includes important protein factors such as mTOR protein, ribosomal protein S6 kinase 1 (S6K1) [11], and eukaryotic initiation factor 4E binding protein 1 (4EBP1) [12]. As an important functional AA and a substrate of protein synthesis, the effect of Phe on protein synthesis through mTOR signaling pathway in animal body remains controversial [13–15]. What is more, the effect of AA, for instance Phe, on pancreatic digestive enzymes synthesis and secretion in dairy cow has not been reported.

The present study hypothesized that Phe could affect the mTOR signaling pathway of PA cells, and then the synthesis of protein enhanced especially those digestive enzymes. The present study was undertaken, therefore, to assess the effect of Phe on the synthesis and secretory response of pancreatic enzymes, amylase, trypsin, lipase in dairy cow. The ultimate goal of present study is to improve the post-ruminal digestion of dairy cow, and to reduce the feed stuff waste as well as the environment pollution of dairy cow industry.

## Materials and methods

This experiment was conducted in the Laboratory of Animal Nutrition at Northwest A&F University (Yangling, China). Two separate experiments were conducted using pancreatic acinar (PA) cells and pancreatic tissue segments (PTS). Both of them came from the pancreatic tissue of dairy calves. All procedures used in this experiment complied with the animal care protocol which was approved by the Northwest A&F University Animal Care and Use Committee.

### Pancreatic tissue preparation

Pancreatic tissue was obtained from three different Holstein calves (79.15 ± 1.68 kg; mean ± SD; 2 months old; weaned). All Holstein calves were raised in the Modern Farming (BaoJi, China). The diet consisted of raw milk and calf starter (Purina, China). The raw materials of calf starter feed included maize, soybean meal, wheat bran, cane molasses, dicalcium phosphate, mountain flour, salt, vitamin A, D3, E. The chemical composition of the starter feed and the concentration of AAs in the milk were shown in Tables 1 and 2, which showed that no additional Phe in calf diets. Calves were fed with milk about 3–4 L/day. The calf starter was added from the third week to eighth week and then calves were slaughtered to collect the pancreatic tissue. All calves were fasted for 12 h before slaughter, then injected with the anesthetic into the jugular vein and bled to death. Three calves were slaughter over 3 days, one calf per day, to provide fresh pancreatic tissues for the culturing. Slaughtering of animals was carried out at the Animal Nutrition Laboratory of Northwest A&F University. Mesentery, fat, and lymph were removed from the pancreas. Approximately 10 g of pancreas was rinsed with ice-cold sterile water and transported in ice-cold saline (0.9% NaCl).

**Table 1:** Chemical composition of the calf starter feed (dry matter basis)

Items	Content (%)
Dry matter	87.06
Crude protein	20.01
Crude ash	15.47
Starch	38.79
NDF	12.20
ADF	6.20
Ca	0.70
Total P	0.38

**Table 2:** Concentration of AAs in the milk^1^ (g/L)

Items	Concentration^2^	Items	Concentration^2^
Arginine	1.429 ± 0.091	Alanine	1.356 ± 0.102
Histidine	0.920 ± 0.055	Aspartate	2.869 ± 0.154
Isoleucine	1.744 ± 0.230	Cysteine	0.358 ± 0.040
Leucine	3.432 ± 0.264	Glutamate	7.605 ± 0.422
Lysine	3.055 ± 0.144	Tyrosine	1.846 ± 0.139
Methionine	0.950 ± 0.142	Glycine	0.838 ± 0.029
Phenylalanine	1.731 ± 0.098	Serine	2.260 ± 0.074
Threonine	1.804 ± 0.059	Proline	6.458 ± 0.307
Valine	2.372 ± 0.149		

1 Milk was from the multiparous Holstein cows.

2 Data are presented as mean ± SD, *n* = 3.

### Acinar cells isolation and primary culture

PA cells were obtained as described [16,17]. Briefly, the procedure involved dairy bull calves pancreatic tissue followed by digesting it in a dissociation medium containing collagenase III (1 mg/ml) in Kreb-Ringer bicarbonate (KRB) [18] buffer with 5% BSA and incubated for 15 min with constant shaking until homogenous solution was obtained. To this, 5 ml of fresh RPMI 1640-Hepes-fetal bovine serum was added and centrifuged at 500 × ***g*** for 30 s. The cell pellet obtained was washed twice followed by centrifugation. Cells were cultured in suspension or in monolayer in RPMI 1640 glutamax medium (Gibco, USA) with supplements (1 nM epidermal growth factor, 5% BSA), 10% fetal bovine serum, penicillin, streptomycin, and soybean trypsin inhibitor and incubated at 37°C with 5% CO_2_.

### Tissues isolation and incubation

The incubation techniques of PTS described were based on similar procedures used for other species or purposes [19,20]. The pancreatic piece was transferred to ice-cold KRB buffer and cut into small segments (approximately 2 × 2 mm, 0.1 g) with a pair of fine scissors. Tissue segments (approximately 100 mg) were blotted dry on a filter paper, weighed using an electronic balance (Mettler Toledo, Shanghai, China), and transferred by rinsing the tissue with 1 ml KRB into 25-ml flasks containing 2 ml of KRB and various substrates (total volume of KRB culture medium = 3 ml). The flasks were gassed with a mixture of 95% O_2_ and 5% CO_2_, capped, and placed in a 39°C shaking water bath at 90 oscillations/min for incubation.

### Treatments and experimental design

**Figure 1:**
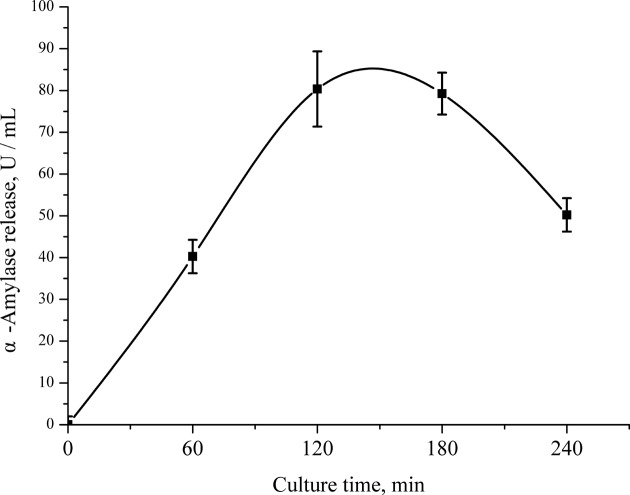
The secretion curve of α-amylase. Influence of culture time on α-amylase release from PA cells. Data are expressed as means ± SEM, *n* = 3.

The isolated PA cells were cultured in six-well cell culture plate and each well had 1×10^6^ cells. All treatment media (complete RPMI 1640 medium) were adjusted to a pH of 7.4, were serum-free, and contained 3.7 mmol non-EAA, 0.70 mM L-arginine, 0.12 mM L-methionine, 0.15 mM L-histidine, 0.42 mM L-isoleucine, 0.45 mM L-leucine, 0.45 mM L-valine, 0.5 mM L-lysine, 0.04 mM L-tryptophan, 0.45 mM L-threonine, 17.5 mmol D-glucose, 0.1 mg insulin, 0.02 mmol phenol red, 0.50 mmol sodium pyruvate, and 14.0 mmol sodium bicarbonate per liter. There were three Phe treatments: the medium containing 0 mM Phe (custom media from Givco, Invitrogen), 0.15 mM Phe (about three times of the arterial concentration of 0.05 mM [21]) or 0.45 mM Phe (about nine times of the arterial concentration). Before and after the culturing, we measured cell viability by trypan blue staining. Only results of more than 95% cell viability were used in the data analysis. In a preliminary experiment, the α-amylase release of PA cells were increased with time and reached the peak at 120 min in complete RPMI 1640 medium, after 180 min, the amylase release was decreased (Figure 1). Based on those results, we chose sampling at 120 min to ensure the maximum secretion of cells. After incubation for 120 min, cells were harvested by scraping in the presence of ice-cold lysis buffer containing 1% (v:v) of protease and phosphatase inhibitors cocktail (Roche, China). Cell lysates from a six-well plate of each medium were combined. The culture medium was also collected for further analysis of enzymes activity. Culturing the cells were repeated 3 days. On each day, cells from a calf were cultured in three six-well culture plates with three kinds of media, respectively. So each Phe treatment had a total of three replicates from three calves (*n* = 3).

PTS incubation experiment was 2 × 3 factorial with two concentration of Phe and three time points as the factors. In our preliminary experiment, 1 g pancreatic tissue was isolated out about 2.4 × 10^7^ acinar cells (Scepter^TM^ 2.0 Cell Counter, Millipore, USA). In this study, we used about 0.1 g pancreatic tissue for incubating. So the number of PA cells in each tissue segment (2.4 × 10^7^) was about 2.4 times the number of PA cells in each well (1 × 10^6^). So the concentration of Phe in PTS medium should be 2.4 times in PA cells medium. The two concentrations of Phe were control (no additions, 0 mM Phe) and 0.35 mM Phe (equivalent to 0.15 mM Phe in PA cells medium). The incubation time was 60 min, 120 min, or 180 min. The experiment was repeated three times on three different days (*n* = 3).

### *In vitro* enzyme release

In order to measure the activity of digestive enzymes in the tissue segments, the supernatant of homogenate was collected after homogenizing. The activity of α-amylase, trypsin, and lipase in the supernatant of homogenate and culture medium (included PA cells culture medium and PTS culture medium) were determined using the commercial kits (Nanjing Jiancheng Bioengineering Institute, China). The enzyme activity was expressed in units per milligram protein (tissue segments homogenate) or units per milliliter (culture medium). One unit was defined as 1 μmol product released per minute at 39°C.

### RNA extraction and real-time quantitative PCR

The expression level of α-amylase, trypsin, and lipase in PA cells and PTS were measured using quantitative real time-PCR (qRT-PCR). The primers for qRT-PCR were designed using the Primer 5.0 design software and NCBI. The sequences of the primers for genes encoding amylase, trypsin, and lipase were listed in Table 3. The qRT-PCR experiment was performed in a total volume of 20 μl using 96-well microwell plates and a Bio-Rad IQ5 Real-Time PCR Detection System. Total RNA was isolated from the cells and tissue segments using method of TRIzol (Takara). RNA quality was determined in Agilent Bioanalyzer and all RNA samples were found to have 260/280 ratios 2.0. For RT-qPCR analysis, total RNA was first converted to cDNA using Takara cDNA transfer kit. cDNA synthesis reactions were carried out in a total volume of 20 μl consisting of 4 μl of 5 × cDNA synthesis buffer, 2 μl dNTP Mix, 1 μl RNA Primer, 1 μl RT Enhancer, 1 μl Verso Enzyme Mix, 1 μl total RNA (1000 ng) sample, and 10 μl molecular grade water. For qRT-PCR, 5 μl of cDNA, 25 μl iTaq Fast SYBR Green Supermix (Takara), and 125 nM of primers were added to each microwell, to a total volume of 20 μl. The PCR was run at 95°C for 3 min, followed by 40 cycles at 95°C for 3 s and 60°C for 30 s. All PCRs were performed in triplicate. The mRNA expression of the target genes were calculated in relevance to the average mRNA expression of a housekeeping gene 18s rRNA of bovine.

**Table 3:** The sequences of the primers

Primer	GenBank number	Sequence (5′–3′)	Base number	Annealing (°C)
Amylase-F	NM_001035016	GAAATGGCCGTGTGACAGAATTTA	24	64.3
Amylase-R		ACAAAGACAAGTGCCCTGTCAGAA	24	
Trypsin-F	NM_001113727	TGTCTGCGGCTCACTGCTAC	20	62.7
Trypsin-R		GCTGGGATGGACGATACTCTTG	22	
Lipase-F	NM_001205820	GTGGAAGCAAATGATGGACAAG	22	61.8
Lipase-R		TGGGTTGAGGGTGAGCAGA	19	
18S rRNA-F	NR_036642	ACCCATTCGAACGTCTGCCCTATT	24	61.2
18S rRNA-R		TCCTTGGATGTGGTAGCCGTTTCT	24	

### Protein preparation and Western blot

Protein concentration in cell lysate and tissue segment homogenate were determined using the BCA assay kit (Thermo Fisher, USA). The samples were boiled at 100°C for 5 min in 5 × sample buffer (CWBIO, China). The protein extracts (60 μg protein each) were electrophoresed in 6–15% SDS-polyacrylamide gels (Bio-Rad, Tichmond, CA). The separated proteins were then transferred onto a nitrocellulose membrane in Tris–glycine buffer containing 20% methanol. The membranes were blocked and immunoblotted with a 1:1000 dilution of a primary antibody including anti-β-actin (CWBIO, China Nos. CW0096M), anti-mTOR, anti-P-mTOR, anti-p70S6K, anti-P-p70S6K, anti-4EBP1, and anti-P-4EBP1 (Cell Signaling Technology, catalog nos. 2972, 2971, 9452, 9459, 9202, and 9205, respectively).

The proteins were detected using either goat anti-rabbit IgG (H+L)–HRP conjugated secondary antibody (1:3000) or goat anti-mouse IgG (H+L) secondary antibody (1 : 5000) with chemiluminescence (ECL) western blot detection reagents. β-Actin was used as an internal control. Western blots were developed and quantified using Image J software. The protein level was quantified by normalizing total and phosphorylated proteins with β-actin.

### Calculations and statistical analysis

Analysis on the amylase release, mRNA expression, and signaling pathway factors phosphorylation of PA cells using one-way ANOVA with SPSS (Version 19.0), Phe treatment was factor and calves as blocks. For the results from PTS, two-way ANOVA procedure was used, Phe treatment and incubation time were factors and calves as blocks. The interaction between Phe and time was included in the model. Protein expression content was calculated as the ratio of the band intensity of β-actin. Differences of *P* < 0.05 were considered significant and data were presented as means ± standard error of the mean (SEM).

## Results

### PA cells

**Figure 2:**
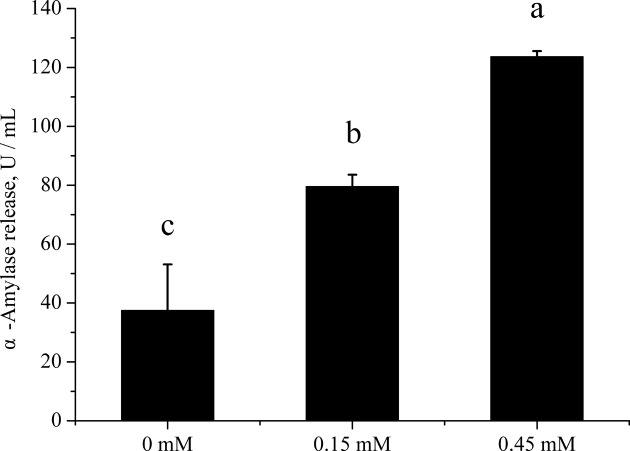
Analysis of α-amylase release of PA cells. Inluence of Phe treatments on α-amylase release of PA cells at 120 min. Data are expressed as means ± SEM, *n* = 3. Different letters mean significantly different (*P* < 0.05).

**Figure 3:**
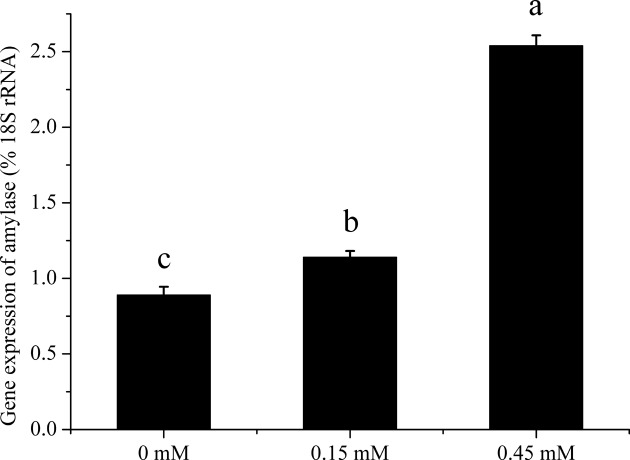
Gene expression analysis of PA cells. Inluence of Phe treatments on α-amylase gene expression of PA cells at 120 min. Data are expressed as means ± SEM, *n* = 3. Different letters mean significantly different (*P* < 0.05).

**Figure 4:**
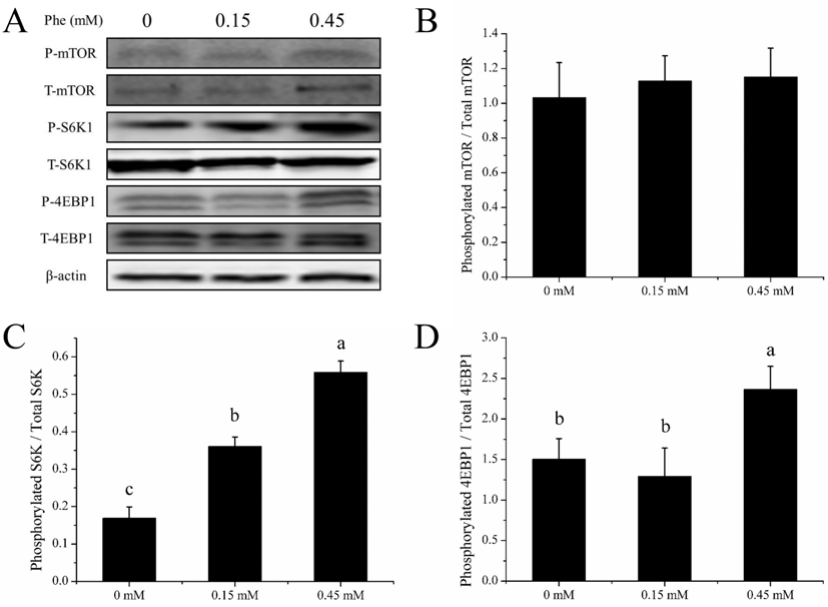
mTOR pathway activity of PA cells. The ratio of phosphorylation to total mTOR signal pathway factors in PA cells of dairy calves cultured at 120 min in the presence of 0–0.45 mM L-phenylalanine. (**A**) The immunoblots of phosphorylation forms and total of mTOR, S6K, 4EBP1. (**B–D**) The ratio of the phosphorylation to total mTOR, S6K, and 4EBP1, respectively. Error bar represents SEM, *n* = 3. Means followed by same or no letter did not differ (*P* > 0.05).

Addition of Phe to PA cells increased (*P* < 0.05) the α-amylase released in culture medium (Figure 2). The lipase activity was very low so we did not measured successfully. The trypsin inhibitor was included in the culture medium, so we also did not measured. Addition of Phe to PA cells increased the gene expression of α-amylase (*P* < 0.05) (Figure 3). The Phe concentration changed from 0 to 0.45 mM did not affect (*P* > 0.05) the ratio of phosphorylation to total mTOR (Figure 4B), but significant increased (*P* < 0.05) the ratio of phosphorylation to total S6K (Figure 4C), and increased (*P* < 0.05) the ratio of phosphorylation to total 4EBP1 (Figure 4D) in high Phe concentration (0.45 mM).

### PTS

**Figure 5:**
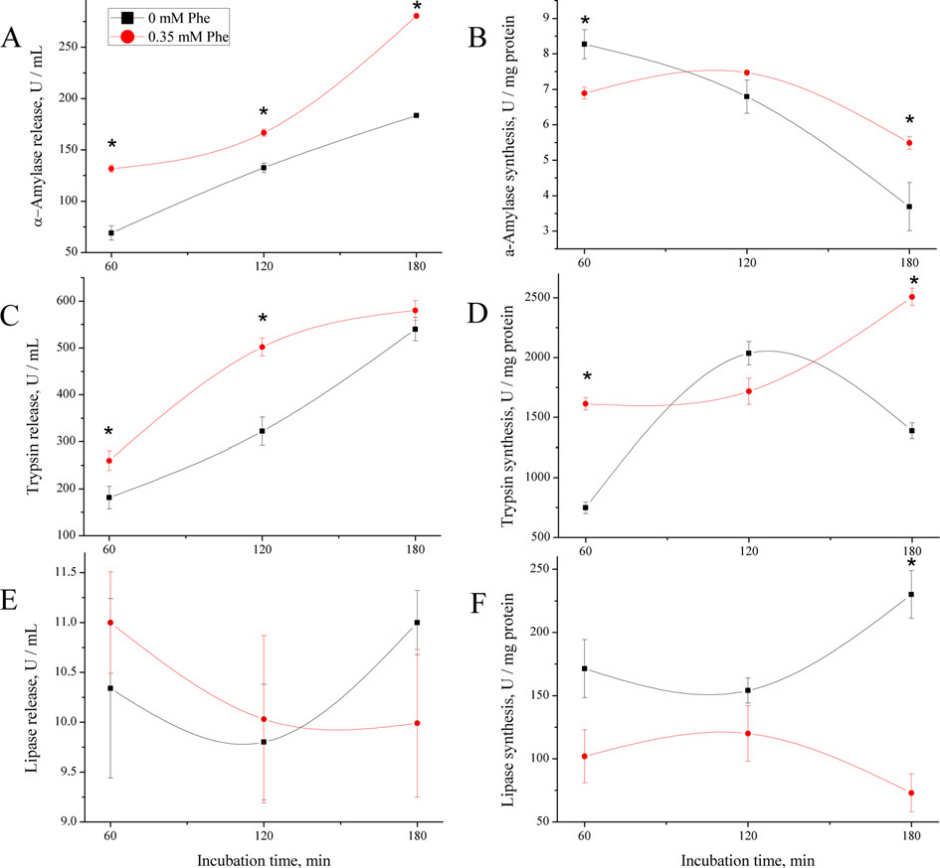
Digestive exzymes synthesis and release analysis of PTS. Inluence of Phe treatments on digestive enzymes synthesis and release of PTS of dairy calves. (**A, C**, and **E**) The synthesis of the α-amylase, trypsin, and lipase respectively in PTS homogenate from 60 min to 180 min. (**B, D**, and **F**) The release of α-amylase, trypsin, and lipase respectively in PTS cultured medium from 60 min to 180 min. Data are expressed as means ± SEM, *n* = 3. * means significantly different (*P* < 0.05).

As shown in Figure 5, the Phe treatment increased pancreatic tissue synthesis of α-amylase (Figure 5A) at 60 min (*P* < 0.05) and 180 min (*P* < 0.05), but not at 120 min (*P* > 0.05). The release of amylase (Figure 5B) increased all the time points in Phe treatment (*P* < 0.05). The synthesis of trypsin (Figure 5C) in Phe treatment was higher at 60 min (*P* < 0.05) and 180 min (*P* < 0.05). The release of trypsin (Figure 5D) increased (*P* < 0.05) at 60 and 120 min in Phe treatment. The lipase synthesis (Figure 5E) was lower in Phe treatment at 60 min (*P* < 0.05) and 180 min (*P* < 0.05). The release of lipase (Figure 5F) was no significant changed (*P* > 0.05).

**Figure 6:**
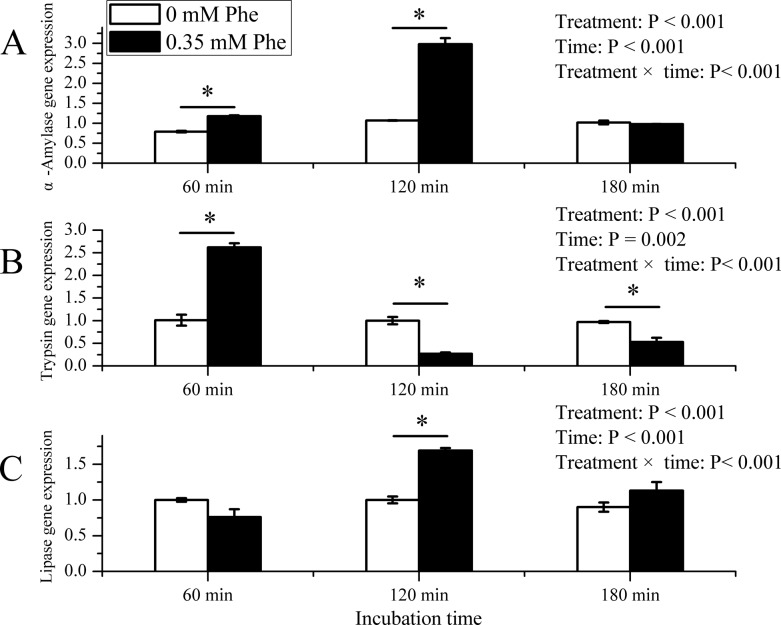
Gene expression analysis of PTS. Inluence of Phe treatments on digestive enzymes gene expression of PTS of dairy calves. (**A**) The α-amylase gene expression. (**B**) The trypsin gene expression. (**C**) The lipase gene expression. Data are expressed as means ± SEM, *n* = 3. ‘—’ means the significant difference between two treatments at one time point. * means significantly different (*P* < 0.05).

As shown in Figure 5, the Phe treatment elevated α-amylase mRNA expression at 60 min (*P* < 0.05) and 120 min (*P* < 0.05), except 180 min (Figure 6A). The gene expression of trypsin gene was enhanced at 60 min (*P* < 0.05), but quite lower at 120 min (*P* < 0.05) and 180 min (*P* < 0.05) in Phe treatment (Figure 6B). As for lipase gene in Phe treatment, the expression was higher at 120 min (*P* < 0.05), but not at 60 min and 180 min (*P* > 0.05) (Figure 6C). There were significant interaction between time and treatment on mRNA expression of enzymes (*P* < 0.001).

**Figure 7:**
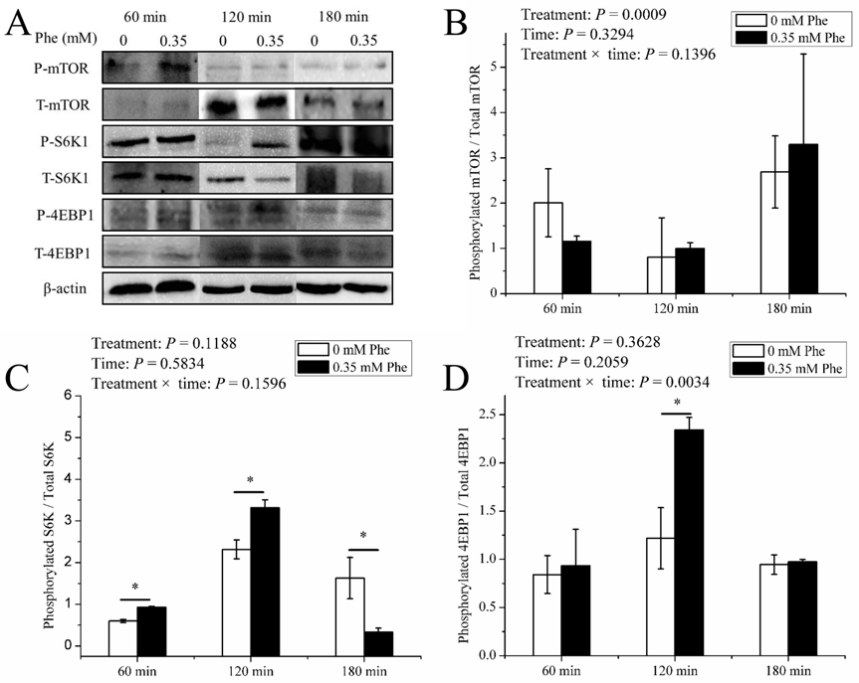
mTOR pathway activity of PTS. The ratio of phosphorylation to total mTOR signal pathway factors in PTS of dairy calves cultured at 180 min in the presence of 0 or 0.35 mM L-phenylalanine. (**A**) The immunoblots of phosphorylation forms and total of mTOR, S6K, 4EBP1. (**B–D**) The ratio of the phosphorylation to total mTOR, S6K, and 4EBP1, respectively. Error bar represents SEM, *n* = 3. ‘—’ means the significant difference between two treatments at one time point. * means significantly different (*P* < 0.05).

The Phe treatment did not affect (*P* > 0.05) the ratio of phosphorylation to total mTOR (Figure 7B) during experiment period. The ratio of phosphorylation to total S6K (Figure 7C) in Phe treatment was higher than control (*P* < 0.05) at 60 min and 120 min, but at 180 min the situation on the contrary. The Phe treatment just increased (*P* < 0.05) the ratio of phosphorylation to total 4EBP1 (Figure 7D) at 120 min. There were significant interaction between time and Phe treatment (*P* = 0.0034) on the phosphorylation of 4EBP1.

## Discussion

Pancreatic α-amylase, trypsin, and lipase initiate hydrolysis, followed by the action of carboxypeptidases A and B [22], which play an important role during digestion process. Meanwhile, pancreatic exocrine protein synthesis is needed for production of secreted digestive enzymes, the growth of pancreas, and the replacement of normal cellular components. In the mature pancreas, 90% of protein synthesis has been estimated to be devoted to a mixture of about 20 digestive enzymes [23]. To match digestive enzyme synthesis to dietary need, synthesis of digestive enzymes also needs to be controlled [24]. In present study, amylase, protease (trypsin), and lipase were measured after PA cells and PTS treated with an extra amount of Phe. It was found in this experiment that Phe could control the enzymes synthesis and release, which was in line with some other researches [8,25]. Digestive efficiency is compromised with the increased dry matter intake (DMI) of a high producing dairy cow; however, limitations to nutrient digestion and absorption are not fully known [22]. The present study could offer a new nutrition strategy to resolve low energy utilization in the small intestine of ruminants. Although our results at the cellular or tissue level showed effective promotion of Phe to pancreatic exocrine enzymes, to transfer these in vitro results to live animals, particularly to supply an effective dose of specific functional AAs such as the Phe to the pancreas of dairy cow, requires further research.

As nutritional strategies are devised to change production efficiency by increasing rumen protect protein, lipid, and perhaps even starch, the digestive system must adapt to utilize these increased nutrient supplies. The first step is to enhance the synthesis and release the digestive enzymes in pancreas of animals. Comparatively little is known about the effects of Phe on pancreatic exocrine secretion in ruminants. Our research team studied the effect of duodenal infusion of Phe for different times on pancreatic exocrine secretion, and found that pancreatic juice was regulated by Phe in long-term infusion (14 days); but nor for short-term (10 h) infusion [8]. In the present study, we found Phe significant increased the synthesis and release of α-amylase in PA cells and α-amylase, trypsin in PTS, but remarkable decreased lipase synthesis in PTS. The literatures showed that an intravenous infusion of AA stimulated pancreatic enzyme secretion in rabbits [26] and in dogs [27]. However, intravenous administration of AA in men did not stimulate pancreatic exocrine secretion [28]. According to Yang et al. [5] wing vein injection of Phe at 0.5 mM level did not stimulate pancreatic amylase secretion under both presence or absence of cholecystokinin (CCK), though pancreatic trypsinogen and chymotrypsinogen outputs were enhanced in chicks. Niederau et al. [29] reported that Phe caused release of both trypsinogen and chymotrypsinogen but not amylase in rat pancreas *in vitro*, so they suggested that the aromatic AA such as Phe are sites of tryptic and chymotryptic cleavage. Our results were similar to these studies. The reason may be differences between species. In future research, we should understand the relationship between AA and enzymes structure.

Regulation of gene expression by AA can occur at any step in the highly specific processes that involve the transfer of information encoded in a gene into its product (RNA and/or protein) [6]. Protein and AAs are necessary as a signal as well as a substrate for pancreatic digestive enzymes synthesis after a meal [25]. Possible mechanisms include changes in tissue mRNA concentrations of enzymes [20]. In our experiment, we observed the level of all kinds of digestive genes changed after the extra Phe was added. In PA cells, α-amylase gene expression increased significantly when Phe concentration increased. The same situation was also shown in PTS experiment. Not only α-amylase, the trypsin also increased rapidly in Phe treatment. The results suggested that Phe may mediate digestive enzyme synthesis by influecing the mRNA translation initiation. In monogastrics, pancreatic enzyme mRNA expression is generally related to the amount of the substrates (protein, triglyceride, and carbohydrate) passing through the small intestine [30].

**Figure 8:**
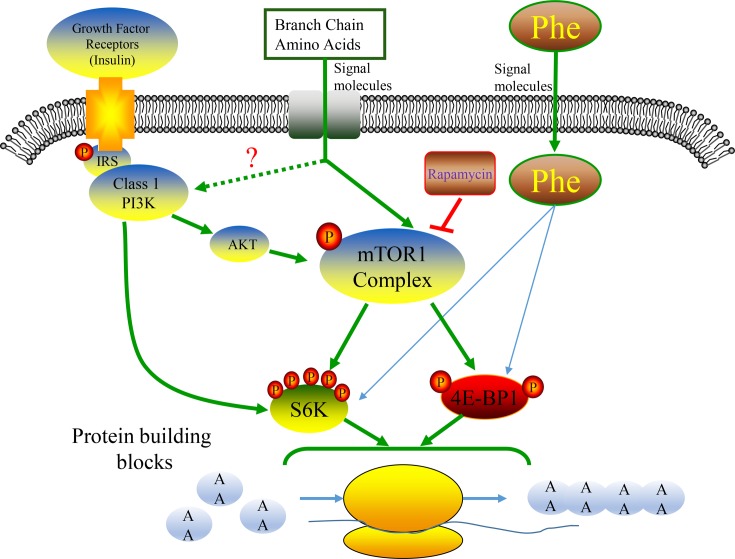
The model of mTOR regulates digestive enzymes synthesis. Proposed model for the synergistic inhibitory effect of Phe on translational mechanisms and protein synthesis in PA cells of dairy calves. We suggest that this effect is conveyed via mTOR signal pathway, especially 4EBP1 and S6K1 phosphorylation.

There is a growing awareness of the need to know the role of AA as signaling molecules in the regulation of protein synthesis [31]. In mammals, the transcription start in mRNA and transcription initiation complex combination. In this biological process, a number of factors are involved, such as S6K, 4EBP1, eIf2α. Besides, the primary pathway being activated in acinar cells is the PI3K-PKB-mTOR pathway [32]. So we focused on the main protein synthesis pathway, the mTOR signal pathway. The results confirmed that Phe is able to regulate the synthesis and release of digestive enzymes both in PA cells and PTSs, and these effects depended on the expression of corresponding mRNA and phosphorylation state of S6K and 4EBP1. The signaling pathway that emerges from the accumulated data on the effects of Phe was illustrated in Figure 8.

It is widely accepted that the mTOR kinase plays a major role in cell growth [33] or protein synthesis [34,35]. mTOR contains two distinct multiprotein complexes, mTORC1 and mTORC2, both differ in their protein composition, regulation, and function [36]. mTORC1 activity is regulated by several upstream pathways, allowing the coordination of cell growth with the levels of energy, growth factors, and nutrients, in particular, AA [37]. Some studies have observed that BCAAs are able to stimulate the mTOR pathway in adipose tissue [38], breast tissue [39], and muscle tissue [40]. However, little is known about the effect of Phe on mTOR signaling pathway. In our experiment, the extra Phe did not affect mTOR phosphorylation both in PA cells and PTSs at the full time range. It was indicated that Phe probably not stimulated mTOR phosphorylation directly. α-Amylase gene and mTOR are main factors for translation; Phe may play an important role in the gene expression, but not affect mTOR.

S6K1, a major downstream target of mTOR, is activated by growth factors such as insulin, and by mTOR, which is itself regulated by AA [41]. S6K1 phosphorylation is usually used as an indicator of mTOR complex 1 activity, which is supported by the observed positive tendency between mTOR phosphorylation and S6K1 phosphorylation [42]. In present study, we observed that extra Phe positively affected S6K1 phosphorylation both in PA cells and PTS. These results were in consistent with skeletal muscle in rats [43] and may indicate that Phe promotes mRNA of digestive enzymes bind to ribosomes via S6K phosphorylation, and then translation start. Phosphorylation of ribosomal protein S6 by p70 S6 kinase, which is believed to be involved in regulating 5′-TOP mRNA translation [44], is one of the earliest events detected following mitogenic stimuli, as part of liver regeneration of following fasting and refeeding. It has been proposed that this phosphorylation increases the affinity of ribosomes for 5′-TOP mRNAs and thus facilitates their initiation [45].

4EBP1 is another major downstream target of mTOR [9,12,46]. Activated mTOR regulates protein translation by directly inducing Pho-4EBP1. Our results revealed that addition of Phe to the medium raised Pho-4EBP1 in high concentration Phe in PA cells and middle time of pancreaic tissue incubation respectively. These results suggested that both the dosage and treatment time can effect the 4EBP1 phosphorylation state. Once being phosphorylated, 4EBP1 releases eukaryotic initiation factor 4E (eIF4E) from the inactive eIF4E·4EBP1 complex to form the active eIF4G·eIF4E complex that binds to mRNA and initiates translation [47]. *In vitro* and *in vivo* studies have shown that other kinases can be implicated in the phosphorylation of 4E-BP1. Mitogen activated protein kinase (MAPK) and casein kinase 2 can phosphorylate purified 4EBP1 [48] and the ERK pathway can regulate this phosphorylation via a mechanism that requires phosphorylation by mTOR as a priming step [49]. In the present study, Phe did not affect mTOR, but S6K1 and 4EBP1 phosphorylation was enhanced, as well as the mRNA expression of α-amylase and trypsin. These results indicate that the regulation of enzymes synthesis was started by the formation of transcription initiation complex.

## Conclusions

Phe could regulate the synthesis and gene expression of digestive enzymes including α-amylase, lipase, and protease, as well as the phosphorylation of S6K and 4EBP1 in pancreas of dairy calves. The mechanism could be that Phe promotes the formation of transcription initiation complex and then the protein synthesis is raised.

## Abbreviations

AA: amino acid
BCAA: branch-chain amino acid
CCK: cholecystokinin
DMI: dry matter intake
EAA: essential amino acid
4EBP1: eukaryotic initiation factor 4E binding protein 1
KRB: Krebs-Ringer bicarbonate
mTOR: mammalian target of rapamycin
PA: pancreatic acinar
Phe: phenylalanine
PTS: pancreatic tissue segments
p70S6K: ribosomal protein S6 protein kinase
S6K1: S6 kinase 1
